# Down-Regulation of Treg Cells and Up-Regulation of Th1/Th2 Cytokine Ratio Were Induced by Polysaccharide from Radix Glycyrrhizae in H22 Hepatocarcinoma Bearing Mice

**DOI:** 10.3390/molecules16108343

**Published:** 2011-09-30

**Authors:** Xiaojuan He, Xiaobing Li, Biao Liu, Li Xu, Hongyan Zhao, Aiping Lu

**Affiliations:** 1 Institute of Basic Research in Clinical Medicine, China Academy of Chinese Medical Sciences, Beijing 100700, China; Email: hxj19@126.com (X.J.H.); 2 School of Basic Medicine, Henan University of Traditional Chinese Medicine, Zhengzhou 450008, China; Email: baishaoyao@163.com; 3 School of Life Sciences, Hubei University, Wuhan 430062, China; Email: 0407130027@163.com; 4 College of Life Science and Technology, Beijing University of Chemical Technology, Beijing 100029, China; Email: xuli_yes@126.com; 5 Institute of Basic Theory, China Academy of Chinese Medical Sciences, Beijing 100700, China; Email: zhaohongyan1997@163.com

**Keywords:** CD4+CD25+ regulatory T cells, Radix Glycyrrhizae polysaccharide, tumor, Th1/Th2 cytokines

## Abstract

Radix Glycyrrhizae polysaccharide (GP) possesses multiple pharmacological activities. However, the effect of GP on CD4+CD25+ regulatory T (Treg) cells has not been elucidated. This study aimed to investigate the effects of GP on Treg cells and Th1/Th2 cytokines in H22 hepatocarcinoma tumor-bearing mice. The results demonstrated that GP inhibits tumor progression. In the lymph nodes of the tumor microenvironment and spleen, the proportion of Treg cells was significantly higher in the tumor-bearing mice. GP administration down-regulated the population of Treg cells (P < 0.01) and decreased lymph node Foxp3 and IL-10 mRNA expression (P < 0.01). In addition, GP treatment decreased IL-10 and TGF-β level (P < 0.01) and increased IL-2 and IL-12p70 level in serum (P < 0.01). In conclusion, GP reduced the proportion of Treg cells and Foxp3 lowered expression in Treg cells, and up-regulated Th1/Th2 cytokine ratio in serum in the tumor bearing mice, which might partially cause the inhibition of tumor growth.

## 1. Introduction

Radix Glycyrrhizae is a commonly used traditional Chinese herbal medicine [1,2]. Radix Glycyrrhizae polysaccharide (GP) is a major active compound extracted from Radix Glycyrrhizae that possesses multiple pharmacological activities. GP modulates macrophage immune functions, induces nitric oxide (NO) production and inducible NO synthase (iNOS) transcription, and reduces oxidative stress in mice [3,4,5].

CD4+CD25+ regulatory T (Treg) cells, which were first identified by Sakaguchi, are a subset of CD4+ T cells with a critical role in the control of antitumor immune responses [6]. In cancer patients, increased levels of Treg cells have been detected in the peripheral blood, in the primary tumor microenvironment, and in draining lymph nodes [7]. Furthermore, the increases of Treg cells are usually associated with the prognosis of cancer patients [8,9]. Treg cells play a role in immunoregulation, mainly by competitively binding IL-2, producing cytokines such as IL-10 and TGF-β, secreting perforin and granzyme, and inducing indoleamine 2,3-dioxygenase (IDO) and B7-H4 expression in antigen-presenting cells (APC) [10]. However, the possible effect of GP on Treg cells is relatively unknown. Therefore, the purpose of this study was to investigate the possible effect of GP on Treg cells and Th1/Th2 cytokines in tumor-bearing mice.

## 2. Results

### 2.1. Effect of GP on Tumor Growth

Tumor weight changes were detected in all tumor-bearing mice. The tumor weight in all of the treatment groups dramatically decreased compared with the untreated control group. The tumor inhibition rate in the mice of the GP, CTX, and GP + CTX groups reached 31.04%, 54.8% and 55.67%, respectively. These results demonstrate that GP treatment affected tumor remission.

### 2.2. Effect of GP on Treg Cells in the Spleen

**Figure 1:**
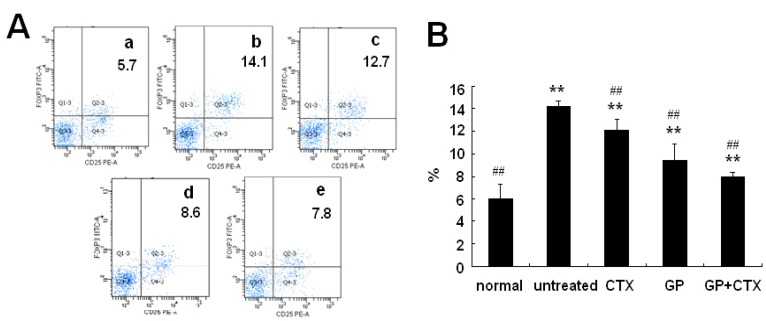
Effects of GP on the proportion of Treg cells in the spleen of tumor-bearing mice. (**A**) Flow cytometric results of the proportion of Treg cells in the spleen. a: Normal control group; b: untreated control group; c: CTX group; d: GP group; and e: GP+CTX group. (**B**) Analysis of the proportion of Treg cells in the spleen. The results are representative of three independent experiments. * P < 0.05, ** P < 0.01, *vs.* the normal control group; # P < 0.05, ## P < 0.01, *vs.* the untreated control group.

As shown in Figure 1, the proportion of Treg cells in the spleen of tumor-bearing mice was higher than that of the normal control mice (P < 0.05). Compared with the untreated control group, GP and/or CTX treatment significantly decreased the proportion of Treg cells (P < 0.05).

### 2.3. Effect of GP on Treg Cells in the Lymph Node

**Figure 2:**
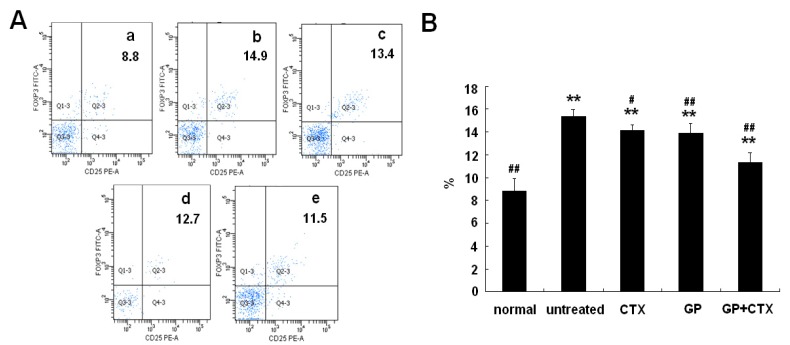
Effects of GP on the proportion of Treg cells in the lymph nodes of the tumor-bearing mice. (**A**) Flow cytometric results of the proportion of Treg cells in the lymph nodes. a: Normal control group; b: untreated control group; c: CTX group; d: GP group; and e: GP+CTX group. (**B**) Analysis of the proportion of Treg cells in the lymph node. The results are representative of three independent experiments. * P < 0.05, ** P < 0.01, *vs.* the normal control group; # P < 0.05, ## P < 0.01, *vs.* the untreated control group.

Lymph node cells in the tumor microenvironment were isolated and analyzed for the presence of Treg cells. As shown in Figure 2, a significant increase of Treg cells was detectable 14 days after tumor inoculation, which agreed with previous research concerning Treg cell expansion in secondary lymphoid organs in the mouse system [11]. After treatment with GP and/or CTX, the proportion of Treg cells decreased significantly (P < 0.05).

### 2.4. Effects of GP on mRNA Expression of Foxp3 and IL-10 in the Lymph Nodes

**Figure 3:**
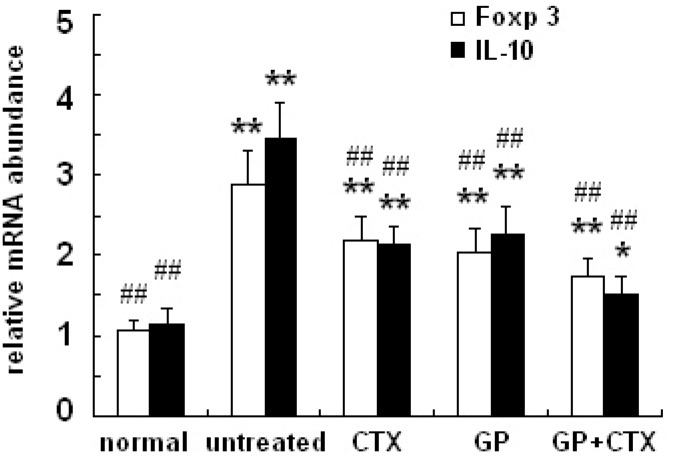
Effects of GP on the mRNA expression of Foxp3 and IL-10 in the lymph nodes. Foxp3 and IL-10 mRNA expressions were determined using real time-PCR. GAPDH was used as the normal control. All experiments were performed three times. * P < 0.05, ** P < 0.01, *vs.* the normal control group; ## P < 0.01, *vs.* the untreated control group.

Foxp3 programs the development and function of Treg cells. IL-10 is an important effector factor of Treg cells. As shown in Figure 3, the expression of Foxp3 and IL-10 mRNA increased significantly in tumor-bearing mice (P < 0.01). After treatment with GP and/or CTX, the mRNA expression of Foxp3 and IL-10 markedly decreased compared with those in the untreated group (P < 0.01).

### 2.5. Effects of GP on the Production of Th1/Th2 Cytokines in Serum

**Figure 4:**
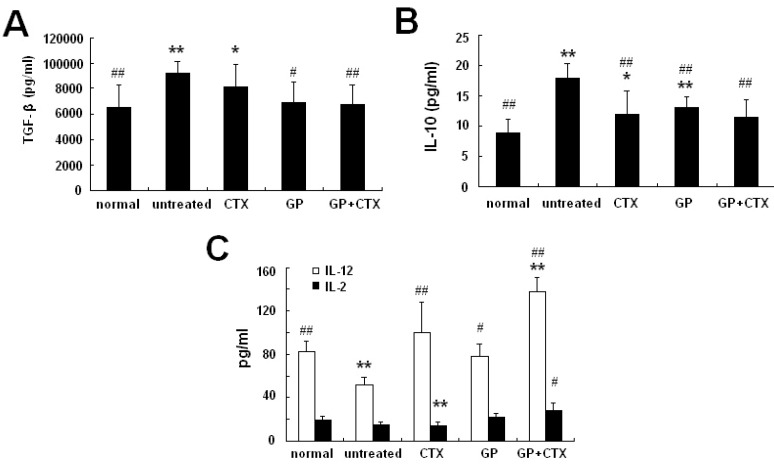
Effects of GP on IL-2, IL-12p70, IL-10 and TGF-β production in the serum of tumor-bearing mice. (**A**) Concentration of TGF-β in serum. (**B**) Concentration of IL-10 in serum. (**C**) Concentration of IL-2 and IL-12p70 in serum. Cytokines were determined using ELISA. All experiments were performed three times. * P < 0.05, ** P < 0.01, *vs.* the normal control group; # P < 0.05, ## P < 0.01, *vs.* the untreated control group.

To determine the Th1 and Th2 cytokine secretion in tumor-bearing mice, levels of IL-2, IL-12p70, IL-10 and TGF-β in the serum were measured. As shown in Figure 4, in tumor-bearing mice, the expression of IL-10 and TGF-β levels were significantly higher than those of normal mice (P < 0.01), whereas the expression of IL-12 was remarkably lower than that of normal mice (P < 0.01). Compared with the untreated control group, GP treatment enhanced the production of IL-12 (P < 0.05) and lowered the production of IL-10 (P < 0.01) and TGF-β (P < 0.05). The combined treatment of GP and CTX increased the serum level of IL-2 (P < 0.05) and IL-12 (P < 0.01) and decreased the serum level of IL-10 and TGF-β (P < 0.01).

## 3. Discussion

Several studies have indicated that GP has numerous biological effects, including anti-adhesive, antioxidant and immunomodulatory activities [3,4,5,12]. However, the function of GP on Treg cells is unknown. In this study, we showed that GP repressed tumor growth (31.04%), decreased the proportion of Treg cells and balanced the Th1/Th2 cytokine levels in tumor-bearing mice. 

Treg cells, a group of negative regulatory cells, suppress the functions of other immune cells. Previous studies have demonstrated that the number of Treg cells in cancer patients is increased and that these Treg cells play a key role in suppressing tumor immunity [10,13,14]. Determining how to effectively remove Treg cells and/or weaken their function has become a new focus in the field of tumor immunotherapy. Our results show that GP significantly decreases the proportion of Treg cells in the spleen and the lymph nodes in tumor-bearing mice and suggests that GP might suppress tumor growth by lowering the proportion of Treg cells.

Foxp3, which is a member of the forkhead/winged-helix family of transcriptional regulators, is a key regulator of Treg cell development and function [15]. Studies have demonstrated that the loss of Foxp3 causes Treg cell deficiencies [16,17]. In addition, Foxp3 regulates the characteristic phenotypes of Treg cells, such as the high expression of CD25, CTLA-4 and GITR and the low expression of CD127 [18]. In the current study, the results show that mRNA expression of Foxp3 in the lymph nodes of GP-treated tumor-bearing mice is decreased. These data indicated that GP-mediated decreases in Foxp3 expression might cause reductions in the proportion of Treg cells in tumor-bearing mice. 

The suppressive activity of Treg cells requires cell-to-cell contact [19]. In addition, several molecules, including IL-10, TGF-β, and granzyme/perforin, are reported to contribute to the suppressive activity of Treg cells [20]. IL-10 has pleiotropic effects on immunoregulation [21] and down-regulates the expression of Th1 cytokines, suppresses the antigen presentation capacity of antigen-presenting cells, and inhibits the activation and effector function of T cells, monocytes and macrophages. TGF-β induces apoptosis [22] and regulates the cell cycle [23] and immune system [24]. TGF-β is important in the regulation of the immune system by Treg cells and the development of Treg cells [25,26,27]. The addition of the anti-TGF antibody or soluble TGF receptor reversed the suppression of Treg cells [27,28,29]. Our data indicated that in tumor-bearing mice, GP significantly decreased IL-10 and TGF-β production in peripheral blood and IL-10 mRNA expression in the lymph nodes. Furthermore, we detected the expression of IL-2 and IL-12p70 in the peripheral blood of tumor-bearing mice. The data showed that GP treatment enhanced the production of IL-2 and IL-12p70. Thus, our results indicate that GP balances Th1/Th2 cytokines levels in tumor-bearing mice, which might be another mechanism for lowering the proportion of Treg cells.

## 4. Experimental

### 4.1. Animals and Cell Lines

Female BALB/c mice (6-8 week of age) were purchased from the Military Medical Science Academy (Beijing, China). Animals were maintained under specific pathogen-free conditions with 12 h-light/dark cycles. All procedures were approved by the Ethical Animal Care and Use Committee at the China Academy of Chinese Medical Sciences. Mouse H22 hepatocarcinoma cells were donated by Dr. Baoqiang Zhao (Institute of Chinese Materia Medica, China Academy of Chinese Medical Sciences) and cultured in DMEM (GIBCO, Carlsbad, CA, USA) with 10% fetal calf serum (FCS).

### 4.2. Drugs

Radix Glycyrrhizae polysaccharide (GP) was purchased from the Beijing office of Pharmagenesis USA (batch No. 20081201). The percentage of polysaccharides in GP was 93.52%. The molecular weights of GP were estimated at 80 kDa based on high performance gel filtration chromatography using pullulans as standards. No endotoxins were detected (<0.10 endotoxin units/mL) in the GP samples using Endospecy.

### 4.3. Animal Treatment

Fifty female BALB/c mice were grouped (n = 10 mice/group) as follows: normal control group, untreated control group, cyclophosphamide (CTX) group, GP group, and GP combined with CTX (GP + CTX) group. Mouse H22 hepatocarcinoma cells (4 × 105 cells in 0.2 mL PBS) were subcutaneously injected into the right axillary region of the BALB/c mice in all groups, except in the normal control group. After 24 h, mice in the normal control group, the untreated control group, the GP group and the GP + CTX group were subcutaneously injected into the napes and backs with different drugs with a volume of 200 μL/mice for 14 days. Mice in the normal control group were given 200 μL PBS; mice in the untreated control group were given 200 μL PBS and mice in the GP group were given 250 mg/(kg•d) GP. Mice in the CTX group and GP + CTX group were intraperitoneally injected with CTX (10 mg/kg•d) for 7 days, and mice in the GP + CTX group were injected with 250 mg/(kg•d) GP. Fourteen days later, the mice were sacrificed. Vein blood was collected from the orbital artery and serum was isolated by centrifugation at 600 × g for 20 min. Axillary and submandibular lymph nodes were separated, and the lymphocytes were collected with mechanical separation. The spleen was removed and flushed with PBS, and splenocytes were isolated by pressing the spleen between the two frosted slides and passed through 48 μm nylonmesh, and the splenocytes were centrifuged once at 260 × g for 5 min and RBCs were removed by 2 mL lysate with 0.84% ammonium chloride. The collected serum, lymphocytes in the lymph nodes in the tumor microenvironment and spleen lymphocytes were stored for further analysis.

### 4.4. Flow Cytometric Analysis

The cells (about 1 × 10^6^) were washed twice with PBS and subsequently labeled with PE/CY5-CD4 and PE-CD25 antibodies (Biolegend, San Diego, CA, USA) for 30 min at 4 °C. After washing twice with cold flow cytometry staining buffer, the cells were incubated with Foxp3 Fix/Perm solution (Biolegend) for 20 min. The cells were washed and stained with the fluorochrome-conjugated anti-Foxp3 antibody (Biolegend) for 30 min. The stained cells were washed and collected using flow cytometry (Becton Dickinson, San Jose, CA, USA) and were analyzed using the CellQuest software package (BD Biosciences, San Jose, CA, USA).

### 4.5. Real-Time PCR Analysis

Total RNA was extracted and dissolved in RNA-free water and quantified using UV-clear microplates (Corning, Beijing, China). A RNA aliquot was verified for its integrity using electrophoresis in a 1% agarose gel that was stained with ethidium bromide. Single-stranded cDNA was synthesized from 2 μg of total RNA using the HiFi-MMLVcDNA kit (Cowin Biotech, Beijing, China). Real-Time PCR was performed using Qiagen Rotor-gene Q and RealSYBR Mixture commercial kits (Cowin Biotech). GAPDH was used as a reference gene. The following primers were used: IL-10, sense, 5’-GCCGGGAAGACAATAACTGC-3’ and antisense, 5’-CCTGGTAGAAGTGA TGCCCC-3’; Foxp3, sense, 5’-AGGAGAAAGCGGATACCA-3’ and antisense, 5’-TGTGAGGACTA CCGAGCC-3’; and GAPDH, sense, 5’-CTCATGACCACAGTCCATGC-3’ and antisense, 5’-CACAT TGGGGGTAGGAACAC-3’. The cycling conditions were 94 °C for 2 min, followed by 40 cycles of 94 °C for 10 min and 60 °C for 30 sec. All samples were measured in triplicate. Differences in gene expression were calculated using the 2–cycle threshold method.

### 4.6. Cytokine Assays

Levels of serum IL-2, IL-10, IL-12p70 and TGF-β were analyzed using a commercially available ELISA kit (Quantikine, R&D Systems, Minneapolis, MN, USA) according to the manufacturers’ instructions.

### 4.7. Statistical Analysis

Statistical analysis was performed using SPSS, version 11.0 (SPSS, Chicago, IL, USA). The data were expressed as the mean ± SD, and significant differences were assessed using Student’s t test. P < 0.05 was considered statistically significant.

## 5. Conclusions

Our data demonstrated that GP reduced the proportion of Treg cells and decreased Foxp3 expression in Treg cells, and up-regulated Th1/Th2 cytokine ratio in serum in H22 hepatocarcinoma tumor bearing mice, which might partially cause the inhibition of tumor growth.
